# Effect of Short-Term Cold Treatment on Carbohydrate Metabolism in Potato Leaves

**DOI:** 10.3390/ijms22137203

**Published:** 2021-07-04

**Authors:** Sławomir Orzechowski, Dorota Sitnicka, Agnieszka Grabowska, Julia Compart, Joerg Fettke, Edyta Zdunek-Zastocka

**Affiliations:** 1 Department of Biochemistry and Microbiology, Institute of Biology, Warsaw University of Life Sciences-SGGW, 02-776 Warsaw, Poland; dorota.sitnicka@wp.pl (D.S.); agnieszka_grabowska@sggw.edu.pl (A.G.); edyta_zdunek_zastocka@sggw.edu.pl (E.Z.-Z.); 2 Biopolymer Analytics, Institute of Biochemistry and Biology, University of Potsdam, Karl-Liebknecht-Str. 24-25 Building 20, 14476 Potsdam-Golm, Germany; compart@uni-potsdam.de (J.C.); fettke@uni-potsdam.de (J.F.)

**Keywords:** cold stress, α-glucan, water dikinase, phosphoglucan water dikinase, chloroplast isolation, glucan phosphorylase, acid invertase

## Abstract

Plants are often challenged by an array of unfavorable environmental conditions. During cold exposure, many changes occur that include, for example, the stabilization of cell membranes, alterations in gene expression and enzyme activities, as well as the accumulation of metabolites. In the presented study, the carbohydrate metabolism was analyzed in the very early response of plants to a low temperature (2 °C) in the leaves of 5-week-old potato plants of the Russet Burbank cultivar during the first 12 h of cold treatment (2 h dark and 10 h light). First, some plant stress indicators were examined and it was shown that short-term cold exposure did not significantly affect the relative water content and chlorophyll content (only after 12 h), but caused an increase in malondialdehyde concentration and a decrease in the expression of *NDA1*, a homolog of the NADH dehydrogenase gene. In addition, it was shown that the content of transitory starch increased transiently in the very early phase of the plant response (3–6 h) to cold treatment, and then its decrease was observed after 12 h. In contrast, soluble sugars such as glucose and fructose were significantly increased only at the end of the light period, where a decrease in sucrose content was observed. The availability of the monosaccharides at constitutively high levels, regardless of the temperature, may delay the response to cold, involving amylolytic starch degradation in chloroplasts. The decrease in starch content, observed in leaves after 12 h of cold exposure, was preceded by a dramatic increase in the transcript levels of the key enzymes of starch degradation initiation, the α-glucan, water dikinase (GWD-EC 2.7.9.4) and the phosphoglucan, water dikinase (PWD-EC 2.7.9.5). The gene expression of both dikinases peaked at 9 h of cold exposure, as analyzed by real-time PCR. Moreover, enhanced activities of the acid invertase as well as of both glucan phosphorylases during exposure to a chilling temperature were observed. However, it was also noticed that during the light phase, there was a general increase in glucan phosphorylase activities for both control and cold-stressed plants irrespective of the temperature. In conclusion, a short-term cold treatment alters the carbohydrate metabolism in the leaves of potato, which leads to an increase in the content of soluble sugars.

## 1. Introduction

Crop plants exposed to cold evolve an array of metabolic changes that allow them to tolerate stress conditions. Exposure of plants to low, nonfreezing temperatures causes changes in physiological, biochemical, and molecular processes. Therefore, metabolic reprogramming refers to the ability of plant cells to alter their metabolism in order to stabilize the new homeostasis [1]. Frost or winter survival is regarded as a complex trait with polygenic inheritance. Two major components of this survival in crop plants are freezing tolerance in the nonacclimated state and cold acclimation capacity. According to the authors, these two main traits associated with frost or winter survival in wild diploid potato species have independent genetic control [2]. Primary cold tolerance is the ability of plants to survive under low-temperature conditions without prior acclimatization and is essential for effectively handling unexpected temperature drops [3]. Among the biochemical changes caused by exposure to cold, the accumulation of soluble sugars [4,5,6,7,8,9] as well as hydrolysis of starch in chloroplasts [10,11] have long been known, but there are still many unresolved challenges. Undoubtedly, sugars that accumulate in plants exposed to cold play many vital roles beyond their primary role of being a source of energy. Soluble sugars can regulate the expression of genes involved in photosynthesis, sugar metabolism, and the synthesis of osmolytes [12]. Therefore, degradation of transitory starch causes the release of soluble sugars and their derivatives, which can be used as respiratory substrates and play important roles in mitigating the stress [13].

Starch phosphorylation by GWD (α-glucan, water dikinase—EC 2.7.9.4) and PWD (phosphoglucan, water dikinase—EC 2.7.9.5) is accepted as the initial step during starch degradation [14]. Diurnal changes in the transcript abundance of PWD in leaves, analyzed using quantitative real-time PCR, are typical for most genes involved in starch degradation in chloroplasts [15]. Furthermore, in *Arabidopsis thaliana*, both GWD and BAM3 (β-amylase EC 3.2.1.2) are considered the most important enzymes involved in starch degradation, both under favorable growth conditions and under cold stress [13]. Maltose (a disaccharide formed from two units of glucose), the main product of starch degradation, can protect proteins, membranes, and the photosynthetic electron transport chain [9] and is further metabolized to sucrose. Sucrose and glucose act as osmolytes to maintain cellular homeostasis. Sucrose is also a precursor in the synthesis of raffinose, which was shown to protect the photosystems in the plastid thylakoid membranes against damage caused by cold [16]. Moreover, carbohydrates can participate in the removal of reactive oxygen species (ROS) generated during stress [17,18], and oxidized sugars formed under stress conditions were detected in vivo in Arabidopsis [19]. Furthermore, overexpression of the *SacB* gene related to the synthesis of fructans led to reduced oxidative damage and has been associated with increased cold tolerance in tobacco [20]. Under stress conditions, sugars may also be involved in cellular signal transduction [12,21,22,23]. More detailed information on the role of sugars during exposure to cold and changes in plant metabolism caused by cold stress can be found in recent reviews [24,25].

Most of the previous studies on physiological and biochemical aspects of the response of potato plants to cold stress were performed with the stress lasting for weeks (resistance phase) [26] or days (acclimatization phase) [27,28,29]. However, according to Kosová and co-workers [30], the first phase of the plant stress response, an alarm phase, lasts usually only for a few hours. Therefore, in this study, we focused on the very early stress response observed in the leaves of potato plants when the plants were exposed to a chilling temperature (2 °C) for a total of 12 h. Under natural conditions, such unexpected temperature drops often occur in the spring in temperate climates. As the earliest effect of chilling temperature on plant cells, a change in the fluidity of their membranes is observed [31]. Then, cold stress reduces the stability of proteins or proteins complexes, triggers the activities of enzymes, such as ROS scavenging enzymes [32], and affects gene expression and protein synthesis [33].

**Figure 1:**
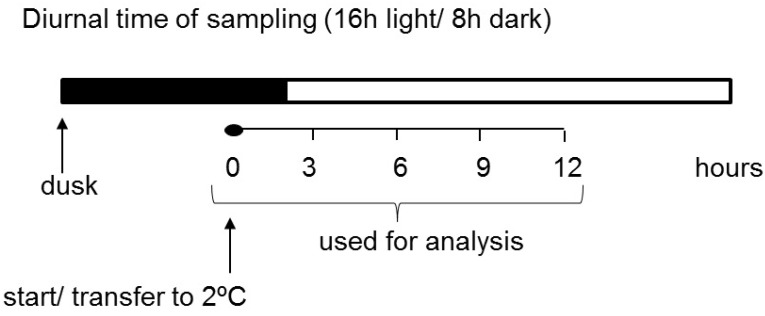
Experimental design of sampling.

Our experiment was designed so that the treatment started two hours before the light phase to decouple the light and temperature parameters from each other (Figure 1). This was done to enable metabolic changes related to cold treatment to be separated from the effects of light alterations. Subsequently, various parameters of carbohydrate metabolism, such as transcript levels, the activity of some important enzymes, and the content of transitory starch and soluble sugars, were investigated in potato leaves for the first time at a very early stage in the plant response to short-term cold treatment. We demonstrated that short-term low temperature has a strong effect on the expression of starch-related dikinases, which are essential for proper starch turnover. After an initially postulated transitory starch degradation, an increased synthesis of starch occurred. Furthermore, the influence of cold temperature on glucan phosphorylase activities, which are believed to play a particularly important role in starch metabolism under abiotic stress conditions, was also shown [7,34,35]. Finally, we verified the hypothesis that, during the early phase of cold stress, there is a significant alteration in carbohydrate metabolism in potato leaves that leads to soluble sugar accumulation.

## 2. Results

### 2.1. The Relative Water Content under Cold Treatment

**Figure 2:**
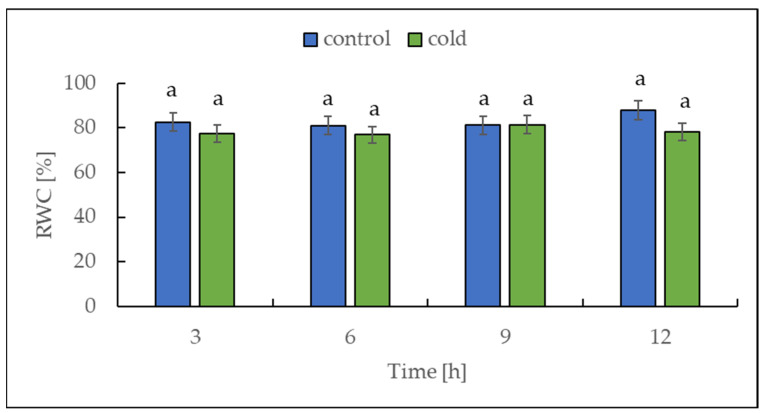
Relative water content (RWC) from potato leaves growing under optimum conditions and exposed to low temperature. The results are the means of at least three biological replicates ± SD. Different letters at the top of the bars indicate significant differences, which were calculated (*p* ≤ 0.05) according to Tukey’s HD (honestly significant difference) post hoc test.

Cold stress (3 °C for 16 h) induced a significant drop in the relative water content (RWC) of tomato plants [36]. For this reason, we decided to measure the RWC in our experiment with potato plants. The relative water content was determined in potato leaves collected from both control and cold-stressed plants at 3 h intervals for 12 h. Over the entire period, no differences in RWC were found between control and cold-treated plants, which is illustrated in Figure 2.

### 2.2. Expression of NADH Dehydrogenase in Cold-Stressed Plants

**Figure 3:**
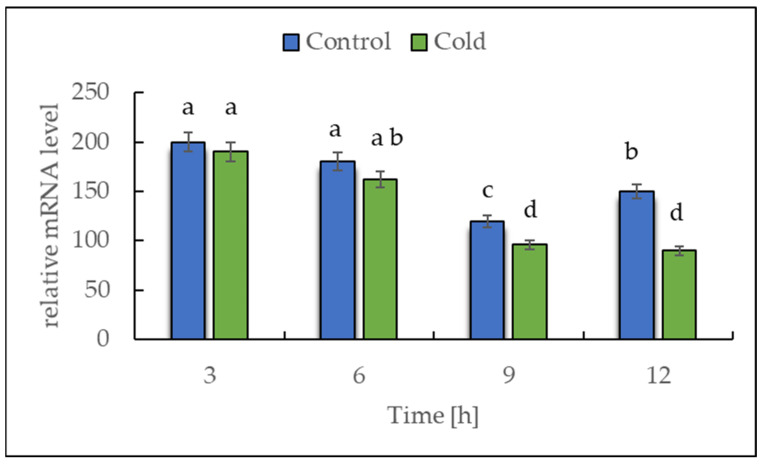
Effect of low temperature on the expression level of *NDA1*, homolog of the gene encoding for NADH dehydrogenase. The results are the means of at least three biological replicates ± SD. Different letters at the top of the bars indicate significant differences, which were calculated (*p* ≤ 0.05) according to Tukey’s HD post hoc test.

The transcript level of *NDA1*, a homolog of the gene encoding for NADH dehydrogenase (EC 7.1.1.2), was investigated by RT-PCR to determine whether the plants showed a rapid response in the first 12 h of exposure to a lower temperature (Figure 3). The expression data analysis revealed that the transcript level of *NDA1* initially remained unchanged, but after 6 h of cold exposure, a continuous, nearly linear, statistically significant decrease was observed.

### 2.3. Malondialdehyde and Chlorophyll Content under Cold Treatment

**Figure 4:**
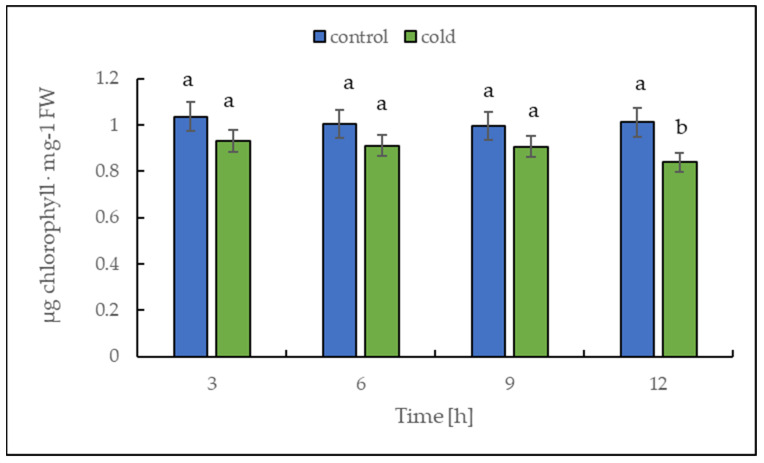
Chlorophyll content calculated on the basis of fresh weight (FW) in potato leaves growing under optimum conditions and exposed to low temperature. The results are the means of at least three biological replicates ± SD. Different letters at the top of the bars indicate significant differences, which were calculated (*p* ≤ 0.05) according to Tukey’s HD post hoc test.

**Figure 5:**
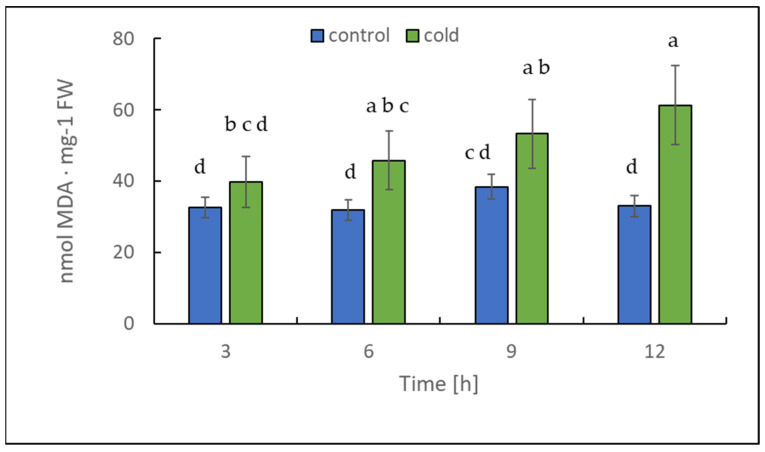
Malondialdehyde (MDA) content calculated on the basis of fresh weight (FW) in potato leaves growing under optimum conditions and exposed to low temperature. The results are the means of at least three biological replicates ± SD. Different letters at the top of the bars indicate significant differences, which were calculated (*p* ≤ 0.05) according to the Tukey’s HD post hoc test.

The chlorophyll content alteration was only statistically significant after 12 h (Figure 4); in contrast, the malondialdehyde content changed significantly in leaves already after 3 h of cold treatment (Figure 5). Our results suggest that the short-term cold treatment did not cause the breakdown of chlorophyll, but quickly led to a change in the structure of lipid membranes in potato leaves.

The level of chlorophyll and malondialdehyde (MDA), an end product of lipid peroxidation [37], can reflect the degree of stress/membrane damage induced by cold stress.

### 2.4. Soluble Sugar Content under Cold Treatment

**Figure 6:**
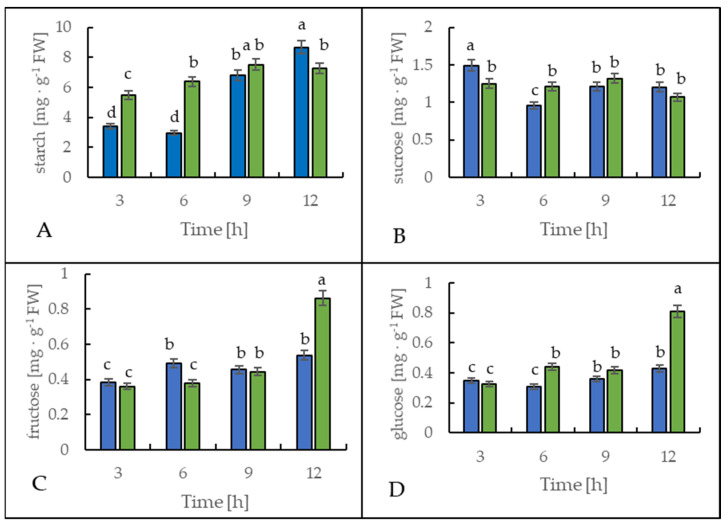
Starch (**A**), sucrose (**B**), fructose (**C**), and glucose (**D**) content in the leaves of potato growing under optimum conditions (blue bars) and exposed to low temperature (green bars). The results are the means of at least three biological replicates ± SD. Different letters at the top of the bars indicate significant differences, which were calculated (*p* ≤ 0.05) according to Tukey’s HD post hoc test.

Figure 6 shows the starch and soluble sugar content in leaves of the potato cultivar Russet Burbank. During the first 6 h, the leaf starch content increased significantly in the cold-treated plants compared to the control plants, as a 130% rise was observed (Figure 6A). However, after 12 h at the end of the light period, the leaves of the cold-stressed plants contained around 20% less starch when compared to the control plants.

The sucrose content in the leaves of both control and stressed plants slightly fluctuated (Figure 6B), but oppositely. In the leaves of the plants exposed to cold, sucrose content was lower by around 30% after 3 h and was higher by around 25% after 6 h of cold treatment than in the control plants.

The glucose and fructose content in the leaves of the cold-exposed plants showed almost no alteration in comparison to the control plants during the first 9 h (Figure 6C,D). However, after 12 h, the content of both monosaccharides rose significantly in the stressed plants. As a result of the lower temperature, glucose and fructose content were 90% or 60% higher than in the control plants, respectively.

### 2.5. Expression of Both StGWD and StPWD under Cold Stress

**Figure 7:**
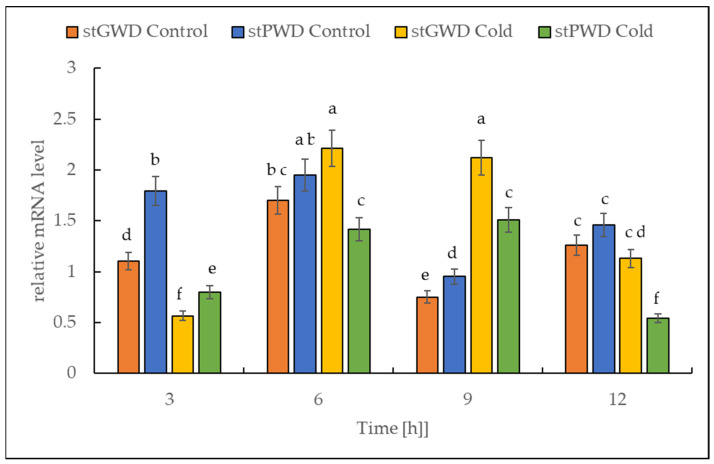
Effect of the low temperature on the expression level of the genes encoding for α-glucan, water dikinases and phosphoglucan, water dikinase in potato leaves. The results are the means of three replicates. Different letters at the top of the bars indicate significant differences, which were calculated (*p* ≤ 0.05) according to Tukey’s HD post hoc test.

The RT-PCR data analysis revealed that under cold treatment, the transcript of both dikinase genes decreased in the first 3 h, followed by a transient increase (Figure 7). The highest level was reached after 9 h. The number of transcripts was higher by almost 300% for StGWD (EC 2.7.9.4) and by 50% for StPWD (EC 2.7.9.5) compared to the initial levels observed at the beginning of the experiment.

### 2.6. Glucan Phosphorylases, Amylases, and the Disproportionating Enzyme 2 Activities under Cold Treatment

**Figure 8:**
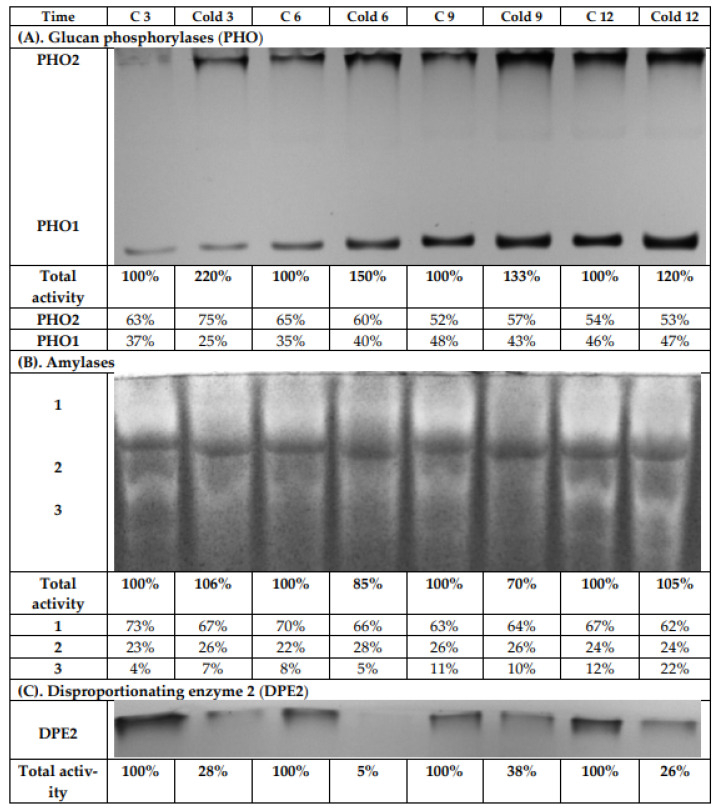
Activity staining of glucan phosphorylases (**A**), amylases (**B**), and disproportioning enzyme 2 (**C**) from leaves of potato growing under optimum conditions or lower temperature (2 °C) after native PAGE. Each lane was loaded with 100 μg of proteins extracted from the leaves of both control and cold-stressed plants after 3, 6, 9, and 12 h. The intensity of the obtained activity bands was quantified by scanning the photos using the software GelQuant.Net 1.8.0 (www.biochemlabsolutions.com, accessed on 3 July 2021). The results were typical of at least four independent experiments.

The leaf extracts from both the control and cold-stressed plants were analyzed using native PAGE and subsequent activity staining to investigate the influence of cold on glucan phosphorylase (EC 2.4.1.1) activities (Figure 8A). After iodine staining, two bands were observed. One band was for the cytosolic phosphorylase isozyme (PHO2), which has a high affinity to glycogen and binds strongly to the immobilized highly branched polymer within the gel. Therefore, it is retained on the top of the separation gel during electrophoresis. The second band can be assigned to the plastidial phosphorylase isozyme (PHO1). It has a low affinity towards glycogen and, consequently, has much greater electrophoretic mobility than PHO2. During the light period, a continuous increase for the enzyme activity for both phosphorylases was observed, so that after 12 h, the highest values were measured. This was found independently for both the control and cold-exposed plants. The greatest total activity increase was observed after 3 h of cold treatment (Figure 8A), and the contribution of PHO2 to the total phosphorylase activity rose after 3 h under low-temperature conditions. However, after 6 h, there was no significant difference between control and stressed plants in the contribution of the isoenzymes to total glucan phosphorylase activity.

Native PAGE and subsequent activity staining was likewise used to investigate the influence of cold on amylase activities (α-amylase, β-amylase, and isoamylase; Figure 8B). After iodine staining, three bands were detected. We observed a decrease in total amylase activities after 6 and 9 h of cold treatment by 15% and 30%, respectively. After 12 h of cold stress, the total amylolytic activity in leaves was the same as in control plants, due to increased activity of the third band, with the greatest mobility in the electric field (Figure 8B).

Another analyzed activity was that of the disproportionating enzyme (DPE; D-enzyme, EC 2.4.1.25). After iodine staining, only one band was visible (Figure 8C), which represented the cytosolic disproportionating enzyme 2 (DPE2). DPE2 catalyzes the transfer of one glucosyl unit from maltose to a heteroglycan, thereby releasing the other glucose moiety [38,39,40,41]. DPE2 has a high affinity towards highly branched glycans, and thus also to glycogen. Therefore, similar to PHO2, it was retained on the top of the separation gel during electrophoresis. A strong decrease (up to more than 70%) in total DPE2 activity was found in stressed plants compared to control plants during the whole experiment. After 6 h cold treatment, the activity of DPE2 in gel was actually negligible. With a prolonged light period, an increase for the DPE2 activity in stressed plants could be observed again (Figure 8C).

**Figure 9:**
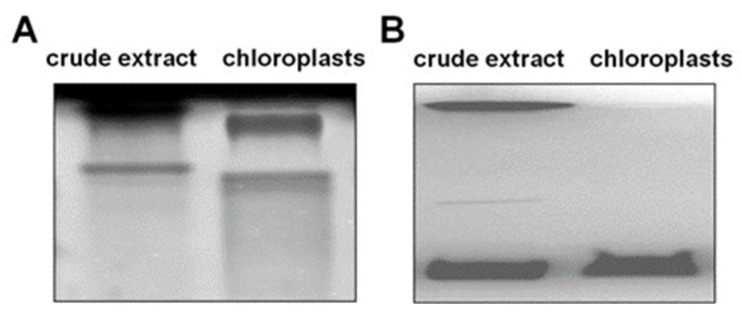
Representative gels of amylolytic (**A**) inverted picture and glucan phosphorylase (**B**) activity in crude extract and chloroplasts isolated from potato leaves. (**A**) Crude extract contains 20 µg proteins; chloroplasts contain 10 µg proteins; (**B**) each lane was loaded with 40 µg of proteins.

To verify the localization of the observed amylase activities, chloroplasts were isolated. All detected amylases were localized in the plastids, as they were also found in the isolated chloroplasts (Figure 9A). However, the zymogram revealed a slight shift in the bands, which was probably the result of different protein compositions, possible protein–protein interactions, or interactions with other cell components. To confirm the purity of isolated chloroplasts, the glucan phosphorylase activity in a gel containing glycogen was determined according to the previous experiment; see Figure 6. In the extracts of isolated chloroplasts, only a band for the plastidial isozyme PHO1 and no contaminating activity of the cytosolic PHO2 was observed (Figure 9B).

### 2.7. The Acid Invertase Activity under Cold Treatment

**Figure 10:**
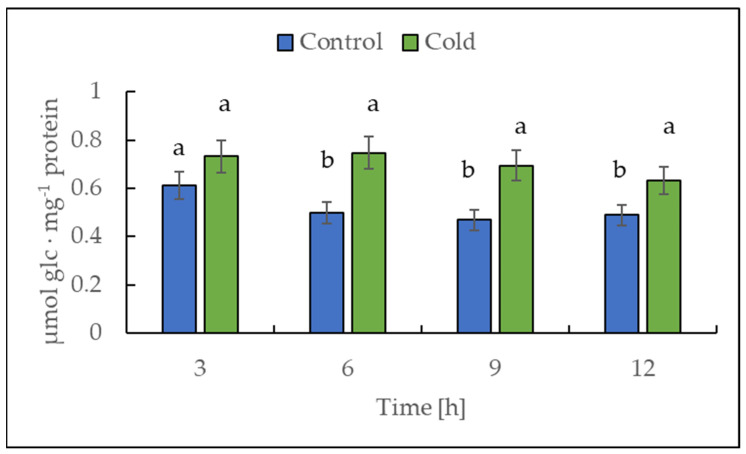
Acid invertase activity in the leaves from potato plants growing under optimum conditions or exposed to low temperature. The results are means of at least three biological replicates ± SD. Different letters at the top of the bars indicate significant differences, which were calculated (*p* ≤ 0.05) according to Tukey’s HD post hoc test.

Cold treatment caused higher acid invertase activity (EC 3.2.1.26). The highest activities were observed after 6 and 9 h of cold stress (Figure 10), with around 50% compared to the control plants.

## 3. Discussion

Sugar sensing and signaling are involved in the control of plant growth and development [42,43,44] as well as in challenging abiotic stresses such as short-term rapid temperature drops. It is nowadays undisputed that the accumulation of sugars and degradation of starch in many plant species have an essential role in cryoprotection and osmoregulation. Our experiment was designed to obtain new insights into short-term cold effects on the carbohydrate metabolism in the leaves of the potato cultivar Russet Burbank. Therefore, the plants were subjected to cold (2 °C) after 7 h of darkness, which was set as point 0. First of all, the effect of chilling temperature on the RWC in leaves was determined (Figure 2). As observed in previous studies in soybeans [45] or *A. thaliana* [46], it could be demonstrated that the RWC is not affected within the first few hours of cold stress, which was also confirmed by our results. To check whether the short-term cold stress has a general effect on the metabolism of plants, NADH dehydrogenase (EC 7.1.1.2) was considered as a stress-marker [47]; therefore, the transcript level of NDA1, homolog of the gene encoding for NADH dehydrogenase, was measured (Figure 3). By extension, the chlorophyll and malondialdehyde content were determined in this context. Although no reduction in the total amount of chlorophyll was found (Figure 4) during the first stage of short-term cold treatment, the malondialdehyde content had strongly increased after 6 h of cold exposure (Figure 5). Demirel and co-workers [48] observed similar results, but after 12 days of drought, in Russet Burbank leaves. Malondialdehyde is a marker for lipid peroxidation and related to oxidative stress and redox signaling [49]. A few hours of cold are enough to start the radical chain reaction, but not to the degree that would lead to severe cell damage and thus to the release of chlorophyll. Likewise, the relative mRNA level of *NDA1*, homolog to the gene encoding for NADH dehydrogenase, had fallen slightly after 9 h of cold stress (Figure 3); according to Van’t Hoff’s rule that biochemical reactions are slowed down by lowering the temperature, a reduction in the expression of this gene has been found. A contrasting result was found for the activities of the two starch-related dikinases, the StGWD and StPWD, after the first 6 h of cold exposure (Figure 7). These results are consistent with a previous report, which demonstrated an increase in the GWD transcript level in Arabidopsis during an early phase of cold acclimation [50]. The dikinases are crucial for proper starch turnover [51]. The phosphorylation alters the surfaces of the granules to such an extent that hydrolyzing enzymes have facilitated access [52]. The elevated transcription levels can be explained by the fact that, under cold conditions, the enzymes act less efficiently, which causes the plants to counteract this and maintain optimal activity by increasing the expression of the corresponding genes. This results in a possible starch degradation that can lead to soluble sugars and thereby to protection of the plant cell. This is consistent with the revealed reduced starch increase observed in the cold-stressed plants after 12 h (Figure 6A). In addition, it has already been shown that starch phosphorylation even takes place in light and that this is indispensable for subsequent degradation during night and may also play a role in synthesis [51]. Therefore, a further explanation would be that the exposure to cold leads to enhanced phosphorylation during starch synthesis. It seems that in the first 6 h during the light phase, starch accumulation might be a result of reduced sink activity because growth retardation at low temperatures is stronger than the reduction of photosynthetic activity [1,24]. Calzadilla and co-workers [53] have noticed an increase in starch granule content in *L. japonicus* chloroplast under low-temperature treatment. After chloroplast isolation, the gel electrophoresis results (Figure 9A) showed that similar bands of amylolytic activity were present in both the crude extracts and in isolated chloroplasts. The purity of isolated chloroplasts was verified by glucan phosphorylase activity staining (Figure 9B). Therefore, it can be concluded that, most likely, under cold conditions, the chloroplastidial amylase activity is not relevant in potato to counteract the abiotic stress factor by degrading starch. The zymograms (Figure 8A–C) showed that short-term cold treatment stimulates the activity of glucan phosphorylase in potato leaves very quickly (Figure 8A), but not amylolytic activity (Figure 8B). Both phosphorylases revealed increasing activity under cold treatment, thus allowing possible starch degradation and also a sugar bypass from the chloroplast to the cytosol by both phosphorylases, as already shown for Arabidopsis [54].

The activity of DPE2, another enzyme involved in soluble carbohydrate metabolism, is strongly reduced by cold stress (Figure 8C). In barley leaves, DPE2 activity was substantially reduced; in contrast, glucan phosphorylases activities were highly increased under salinity stress. The authors suggest different responses of DPE2, PHO1, and PHO2 in the maintenance of leaf starch concentration during salinity stress [32]. The elevated activity of the acid invertase after the first 3 h of cold treatment could indicate that not exclusively starch but possibly sucrose is broken down in a rapid metabolic response in Russet Burbank leaves (Figure 10), which would prevent the accumulation of sucrose in the vacuoles [55]. Moreover, sucrose could be used to receive a precursor for the synthesis of specific compounds, such as an increase in glucose and fructose content, to limit the adverse effects of cold [56]. The sucrose content fluctuated and was only after 3 and 12 h significantly lower than in control plants (Figure 6C). As already mentioned for the dikinases, the lower temperature could lead to reduced activity of the acid invertase, which is compensated by the plant through increased enzyme synthesis to obtain the optimal level of efficiency [3]. However, the analysis of soluble sugars including glucose and fructose disclosed the accumulation of monosaccharides in response to cold after a lag phase at 12 h (Figure 6A,B), which could be a result of the decline in the sucrose content (Figure 6C) and is consistent with former studies [9,50]. The high level of soluble sugars in the cell might serve as an osmoprotectant. In addition, the involvement of glucan phosphorylases in starch metabolism in response to abiotic stresses could be shown several times [4,31,57], so the activity was also examined for the potato cultivar used in the leaves in this study (Figure 8A). On the one hand, an increase in total phosphorylase activity was found during cold exposure, but on the other hand, a general enhancement in activity in the course of the light phase was observed, both for the plants exposed to cold as well as for the control plants. The contributions of the phosphorylases change during the light period. At the beginning of the light period, around two thirds of the detected activity was due to the cytosolic phosphorylase (PHO2), but at the end of the light period, the contribution of both PHO1 and PHO2 was very similar and approximately 50%. The conditions for the detection of both phosphorylases used here overestimate the cytosolic isoform; however, this overestimation is constant for all time points and, thus, the detected alteration of the ratio is real, even the percent values are dependent on the detection system.

These data indicate that the glucan phosphorylase activities and their functional role depend on the environment, generally from the time of day and then particularly from the temperature, which suggests their involvement in the regulation of the plant response to cold. It has already been demonstrated that PHO1 plays a crucial role in starch synthesis, especially during cold. The enzyme is able to maintain activity at low temperatures and shows significantly increased substrate affinity under these conditions [4,57,58]. In contrast, there are indications that the cytosolic PHO2 is part of the net starch breakdown pathway [59]. Based on the current studies of cold-induced changes in carbohydrate metabolism, the plant response may be divided into the following three stages: an early response to the displacement of homeostasis lasting several hours—an alarm phase; an intermediate stage, including the reprogramming of central sugar metabolism approximately 12 to 24 h after the beginning of cold; and the late response after 24 h, during which the stabilization of a new state of metabolic homeostasis with respect to carbohydrate metabolism is established [9,60]. Currently, little is known about the adaptation mechanisms of plants that are permanently subjected to temperature alterations caused by rapid shifts in weather, seasons, and by climate changes, so further effort is required to obtain a more precise picture of these processes.

## 4. Materials and Methods

### 4.1. Plant Material and Cold Treatment

Plants of the potato (*Solanum tuberosum* L.) cultivar Russet Burbank were grown in 12 L pots, filled with commercially available light horticultural soil, and were watered twice a week. The plants were cultivated in a growth chamber with a 16 h photoperiod (day/night temperatures of 22/15 °C), 50% relative humidity, and a midday photosynthetic photon flux density of approximately 200 μmol m^−2^ s^−1^.

When the plants were 5 weeks old, half of them (five plants) were moved to low-temperature conditions (2 °C). The cold treatment was started 1 h before light, which was set as point 0 (Figure 1). Then, medium-sized leaves were collected from both stressed and control plants at 3 h intervals for a total of 12 h. The samples were frozen in liquid nitrogen and stored at −80 °C until analysis. The experiment was repeated three times.

### 4.2. Relative Water Content (RWC)

Relative water content was estimated in three leaves per sampling time and used as a reference to evaluate the water status of plants according to Nevyl and Battaglia [46]. RWC was measured on fully expanded leaves. Three biological replicates were collected from separate plants. The fresh weight of harvested leaves was immediately measured; then, the leaves were incubated overnight in distillate water at room temperature and in darkness. Excess water was removed by blotting with tissue paper and the weight of turgid tissues was recorded. Then, turgid leaf samples were dried in a drying oven at 110 °C overnight. Finally, dried leaf sample weights were recorded. RWC values were calculated using the following equation: RWC (%) = [(fresh weight- dry weight)/(turgid weight-dry weight)] × 100.

### 4.3. RNA Extraction

Total RNA was isolated from the leaves of control and cold-stressed potato plants using the NucleoSpin RNA Plant Kit (Macherey-Nagel, Düren, Germany). RNA quantity and purity were determined spectrophotometrically while RNA integrity was evaluated after electrophoresis in a 1.3% (*w*/*v*) agarose gel.

### 4.4. Two-Step Real-Time PCR

One microgram of each RNA sample was reverse-transcribed into cDNA using the Transcriptor First Strand cDNA Synthesis Kit (Roche) and oligo(dT)18 primers according to the manufacturer’s instructions. Synthesis of cDNA was performed from three independent RNA extractions for each time point to obtain biological replicates. Based on the sequence of potato GWD (GenBank EU599037) and PWD (GenBank GU045560), two pairs of primers were designed. The sequences of the forward and reverse primers used in the RT-PCR reaction were as follows: GWD- F: 5′ CCC ACG ATC TTA GTA GCA AA 3′, R 5′ TTA GCT CCA ACC ATT TCA CT 3′ and PWD- F: 5′ CAA TAG CTA TGC GTC GGA AGT G 3′, R: 5′ GCT TTG CAT TCC TCG GGC TTC 3′. RT-PCR was performed using the LightCycler FastStart DNA Master SYBR Green I Kit (Roche Diagnostics, Indianapolis, IN, USA) and 250 nM of forward and reverse primers in a Light Cycler 2.0 device (Roche Diagnostics) according to the manufacturer’s instructions. PCR conditions were as follows: initial denaturation at 95 °C for 10 min, followed by 40 cycles of 95 °C for 10 s, 62 °C for 5 s, and 72 °C for 12 s. The relative gene expression was evaluated with LightCycler 4.1 software using a comparative ratio of the examined gene over the gene encoding elongation factor 1-α (EF1-α; GenBank AB061263) and β-tubulin (GenBank 609267), which were used as the reference genes and were amplified using primers designed by Nicot et al. [61].

### 4.5. Carbohydrate Analysis

For sugar extraction, 50 mg of leaf tissue was ground into a fine powder in liquid nitrogen using a mortar and pestle. Soluble sugars were extracted three times with 1 mL of 80% (*v*/*v*) ethanol by heating at 80 °C for 30 min. After each heating, the samples were cooled and centrifuged for 10 min at 4 °C with 16,000× *g*. The supernatants obtained after each centrifugation were combined and the ethanol was evaporated. The dried residues from the soluble fractions were then resuspended in 0.5 mL of distilled water and used for soluble sugar determination. The pellet was dried at 60 °C and stored until further analysis of starch. The content of glucose, fructose, and sucrose was determined enzymatically according to Bergmeyer et al. [62] and Jones et al. [63] by the gradual addition of hexokinase (EC 2.7.1.1), phosphoglucose isomerase (EC 5.3.1.9), and invertase (EC 3.2.1.26) in the presence of glucose-6-phosphate dehydrogenase (EC 1.1.1.49) (Sigma-Aldrich, St. Louis, MO, USA). The reaction mixture with a total volume of 0.7 mL contained the following: 100 mM imidazol, pH 6.9, 0.5 mM MgCl_2_, 1.5 mM ATP, 1 mM NADP^+^, 0.5 mU glucose-6-phosphate dehydrogenase, and 10–25 µL of the soluble sugar extract. After 5 min of preincubation at 30 °C, the soluble sugars were quantified photometrically by measuring the change in absorbance at 340 nm after the addition of each enzyme. First, 0.5 mU hexokinase was added for glucose determination; then, 2 mU phosphoglucose isomerase for calculation of the fructose content; finally, 125 U invertase for sucrose determination. Each sample was tested in duplicate. Starch was quantified using the Megazyme protocol (Megazyme Total Starch Assay Procedure, AOAC method 996.11; Megazyme, Bray, Ireland). The dried pellet was resuspended in 0.3 mL of dimethylsulfoxide (DMSO), vortexed vigorously, and boiled at 100 °C for 15 min to facilitate gelatinization of the starch. The enzymatic hydrolysis was performed using a thermostable α-amylase and glucoamylase (Megazyme, Bray, Ireland). A volume of 0.45 mL (340 U) α-amylase in 50 mM MOPS buffer, pH 7.0, was added and incubated at 100 °C for 15 min. The sample was then cooled down, and 0.6 mL of 200 mM sodium acetate buffer (pH 4.7) and 15 μL of glucoamylase (50 U) were added. The sample was then incubated for 30 min at 50 °C, centrifuged for 10 min at 4 °C with 16,000× *g*, and the supernatant was used for indirect starch quantification using the Somogyi–Nelson method [64,65]. Each sample was tested in duplicate.

### 4.6. Chloroplast Isolation

Chloroplasts were isolated according to Rödiger et al. [66], with minor modifications. First, 10 g of potato leaves were harvested after the first hour of the day and directly homogenized in a blender in 60 mL of ice-cold extraction buffer (15 mM MOPS, pH 7.4, 0.45 M sucrose, 1.5 mM EDTA, 0.6% (*w*/*v*) PVP insoluble, 0.2% (*w*/*v*) BSA, 10 mM DTT, 0.2 mM PMSF). For the step gradient, Percoll was diluted in sorbitol resuspension buffer (50 mM HEPES, pH 8.0, 0.33 M sorbitol). Intact chloroplasts, which accumulate at the interphase between 35% (*v*/*v*) and 80% (*v*/*v*) Percoll, were finally resuspended in 5 mL of sorbitol resuspension buffer. After 15 min of centrifugation at 3000× *g* at 4 °C, the chloroplasts in the pellet were lysed in 0.5 mL 50 mM Tris-HCl, pH 8.0, 5 mM EDTA.

### 4.7. Leaf Crude Extract Preparation

The samples of 0.5 g of potato leaves were homogenized in a mortar with 1 mL of 50 mM Tris-HCl, pH 8.0, 5 mM EDTA and centrifuged for 15 min at 16,000× *g* at 4 °C.

### 4.8. Determination of Chlorophyll, Malondialdehyde, and Protein Content

Total chlorophyll content in leaf tissues (100 mg) was estimated using the dimethylsulfoxide (DMSO) method, as described by Hiscox and Israelstam [67]. Absorbance of the extract was taken at 649 and 665 nm against the DMSO blank using a spectrophotometer. Total chlorophyll content was calculated on the basis of fresh weight (FW). The amount of malondialdehyde (MDA) was measured according to the thiobarbituric acid test [34]. Tissue samples of 150 mg were homogenized with 1.5 mL 0.1 M potassium phosphate buffer, pH 6.8, and were centrifuged for 15 min at 10,000× *g* and 4 °C. Supernatants were mixed with TBA (0.5 % thiobarbituric acid in 15% trichloroacetic acid) in 1:1 proportions and incubated for 20 min at 100 °C. After cooling, the solutions were centrifuged for 10 min at 10,000× *g* and the absorbance was measured at 532 nm and 600 nm. The total protein content in isolated chloroplasts and the leaf crude extracts was quantified according to Bradford et al. [68], with bovine serum albumin as a standard.

### 4.9. Amylase, Glucan Phosphorylase, and Disproportioning Enzyme 2 Zymography

The activity of amylases and phosphorylases was determined in gel after separation of proteins by electrophoresis under nondenaturing conditions. Native discontinuous PAGE (polyacrylamide gel electrophoresis) was performed in 8% separating gels containing 0.05% soluble starch (amylase) or 0.1% glycogen (glucan phosphorylase and disproportioning enzyme) and in 4% stacking gels as described by Laemmli et al. [69], without SDS. Electrophoresis was carried out at 200 V for 3.5 h at 4 °C in 0.2 M Tris-glycine buffer, pH 9.1. For determination of amylase activity, the gel was washed twice in 0.2 M sodium acetate, pH 4.5, 2.5 mM MgCl_2_, 2.5 mM CaCl_2_ for 15 min and then incubated in this buffer overnight at 25 °C. Glucan phosphorylase activity staining was performed as described by Fettke et al. [57], except that the gels were incubated for 1 h (the cold treatment experiment) or 20 h (the chloroplast isolation experiment). The disproportioning enzyme 2 activity was measured similarly to glucan phosphorylase activity, except that the gels were incubated with 10 mM maltose and not with glucose-1-phosphate. After incubation, the gels were stained with Lugol solution (1% (*w*/*v*) KI and 0.25% (*w*/*v*) I_2_).

### 4.10. Measurement of Acid Invertase Activity

Plant tissues were homogenized in 50 mM HEPES-KOH, pH 7.4, 5 mM MgCl_2_, 1 mM EDTA, 1 mM EGTA, 1 mM PMSF, 5 mM DTT, 0.1% (*v*/*v*) Triton-X-100, and 10% (*w*/*v*) glycerol. The suspension was centrifuged at 16,000× *g* for 15 min at 4 °C. Acid invertase was assayed in 50 mM sodium acetate buffer with pH 4.7 using 100 mM sucrose as a substrate and 25 µL of extract in a final volume of 0.25 mL. The glucose and fructose contents were determined according to the method described by Lindsay et al. [70]. Briefly, the reaction was performed for 30 min at 30 °C and stopped by the addition of 0.25 mL 1% (*w*/*v*) 3,5-dinitrosalicylic acid sodium salt (DNS). Then, the mixture was incubated for 10 min at 100 °C, cooled on ice, and diluted with 1 mL H_2_O before photometric measurements at 540 nm. Acid invertase activity was calculated based on the standard curve made after incubation of DNS with glucose and fructose mixture in equimolar concentrations. 

### 4.11. Statistical Analyses

All data were analyzed using two-way ANOVA and Tukey’s HD (honestly significant difference) post hoc test to assess the main effects of treatments and time as the independent variables [71]. The level of significance was set at α = 0.05.

## Data Availability

Not applicable.

## Abbreviations

BAM	Beta-amylase
DPE	Disproportionating enzyme
GWD	Glucan, water dikinase
PHO	Glucan phosphorylase
NDA1	Homolog of NADH dehydrogenase
MDA	Malondialdehyde
PWD	Phosphoglucan, water dikinase
